# *Streptococcus suis* Meningitis: A Systematic Review and Meta-analysis

**DOI:** 10.1371/journal.pntd.0004191

**Published:** 2015-10-27

**Authors:** Anusha van Samkar, Matthijs C. Brouwer, Constance Schultsz, Arie van der Ende, Diederik van de Beek

**Affiliations:** 1 Academic Medical Center, Center of Infection and Immunity Amsterdam (CINIMA), Department of Neurology, Amsterdam, the Netherlands; 2 Academic Medical Center, Department of Global Health-Amsterdam Institute for Global Health and Development, Amsterdam, the Netherlands; 3 Academic Medical Center, Center of Infection and Immunity Amsterdam (CINIMA), Department of Medical Microbiology, Amsterdam, the Netherlands; 4 Netherlands Reference Laboratory for Bacterial Meningitis, Academic Medical Center, Amsterdam, the Netherlands; University of Tennessee, UNITED STATES

## Abstract

**Background:**

*Streptococcus suis* is the most common cause of meningitis in pork consuming and pig rearing countries in South-East Asia. We performed a systematic review of studies on *S*. *suis* meningitis to define the clinical characteristics, predisposing factors and outcome.

**Methodology:**

Studies published between January 1, 1980 and August 1, 2015 were identified from main literature databases and reference lists. Studies were included if they were written in West-European languages and described at least 5 adult patients with *S*. *suis* meningitis in whom at least one clinical characteristic was described.

**Findings:**

We identified 913 patients with *S*. *suis* meningitis included in 24 studies between 1980 and 2015. The mean age was 49 years and 581 of 711 patients were male (82%). Exposure to pigs or pork was present in 395 of 648 patients (61%) while other predisposing factors were less common. 514 of 528 patients presented with fever (97%), 429 of 451 with headache (95%), 462 of 496 with neck stiffness (93%) and 78 of 384 patients (20%) had a skin injury in the presence of pig/pork contact. The case fatality rate was 2.9% and hearing loss was a common sequel occurring in 259 of 489 patients (53%). Treatment included dexamethasone in 157 of 300 (52%) of patients and was associated with reduced hearing loss in *S*. *suis* meningitis patients included in a randomized controlled trial.

**Conclusion:**

*S*. *suis* meningitis has a clear association with pig and pork contact. Mortality is low, but hearing loss occurs frequently. Dexamethasone was shown to reduce hearing loss.

## Author Summary

Meningitis is a common manifestation of *Streptococcus suis* infection. *S*. *suis* is endemic in pork consuming and pig rearing countries. We systematically reviewed the clinical characteristics, predisposing factors and outcome of *S*. *suis* meningitis. We identified 913 patients included in 24 studies, with a mean age of 49 years and a majority of male patients (82%). Exposure to pigs or pork was present in 61%, with a skin injury being present in 20%. Fever was present in 97% of patients, headache in 95%, neck stiffness in 93%. The mortality was 2.9% and hearing loss was common occurring in 53% of patients. Dexamethasone was associated with reduced hearing loss.

## Introduction

Bacterial meningitis is a severe infectious disease with a high mortality and morbidity. The estimated incidence is 2.6–6 per 100,000 adults per year in developed countries and several times higher in low-income settings [1]. Most pathogens causing bacterial meningitis are transmitted between humans (*e*.*g*., *Streptococcus pneumoniae* and *Neisseria meningitidis*), while others can be acquired through food ingestion (*e*.*g*., *Listeria monocytogenes*) [1, 2]. Transmission of pathogens causing bacterial meningitis can also occur directly from animals to humans, a condition referred to as zoonotic bacterial meningitis.

One of the most common zoonotic pathogens causing bacterial meningitis is *Streptococcus suis*. This pathogen has its natural reservoir in pigs and may cause meningitis, endocarditis and sepsis in humans after contact with pig or pork [3, 4]. Due to high pork consumption and frequent small scale pig rearing, *S*. *suis* infection is endemic in South-East Asia, where several outbreaks and cohort studies of *S*. *suis* meningitis have been reported [5–8]. Nevertheless, cases of *S*. *suis* meningitis occur all over the world [9], particularly in patients having occupational contact with pigs or pork, such as abattoir workers and butchers [10]. The clinical manifestations, epidemiology and outcome of *S*. *suis* infection in humans were described in a recent systematic review and meta-analysis [9]. This review included studies through 2012 and did not review characteristics of *S*. *suis* meningitis separately (the condition comprised 68% of cases). We performed a systematic review on studies on *S*. *suis* meningitis to define the clinical characteristics, risk factors and outcome of *S*. *suis* meningitis.

## Methods

We searched the main databases (PubMed, ScienceDirect, Google scholar) for published articles describing cases of *S*. *suis* meningitis, published from January 1980 to August 2015. We used the search terms “*Streptococcus suis* AND meningitis”, and searched the literature for cohort studies using the term “*Streptococcus suis*”. We also searched the reference lists of the articles identified by this search strategy and selected those that we judged to be relevant. Articles written in English, Dutch, French, German, Spanish, Italian and Portuguese were included. Articles describing at least 5 patients with *S*. *suis* meningitis were included if at least one clinical characteristics or ancillary investigation was described, unless no sub-analysis for *S*. *suis* meningitis was performed (e.g. *S*. *suis* infection or streptococcal meningitis).

All articles meeting the inclusion criteria were read and systematically processed into a database of clinical data. The variables were as follows: patient characteristics, predisposing factors, clinical presentation, ancillary investigations, and outcome. Predisposing factors were defined as 1) Contact with pigs or pork, defined as preparing pork, consumption of raw pork or other swine materials (e.g. raw pig blood), occupations related to pigs or pork (e.g. abattoir workers, butchers), or breeding pigs at home [4], and/or 2) An immunocompromised status for bacterial meningitis caused by infection with Human Immunodeficiency Virus (HIV), a history of immunosuppressive medication, cancer, splenectomy, or alcoholism [2]. When patients were reported to be ‘not immunocompromised’, we assumed no immunosuppressive medication, splenectomy or HIV-infection in these patients. Skin injury was defined as cuts or scrapes, since skin rash could be misidentified as bruises (as seen in meningococcal sepsis).

As data description was heterogeneous between studies, all data are presented as number for which a characteristic was present out of the total number for which the characteristic was evaluated. We described the relevant characteristics using proportions with 95% confidence intervals (CIs) for categorical factors (sex, predisposing factors), and mean with standard deviation (SD) for continuous factors (age, laboratory parameters). For the latter, medians were converted to means by using proposed formulas [11].

## Results

### Study characteristics

**Fig 1:**
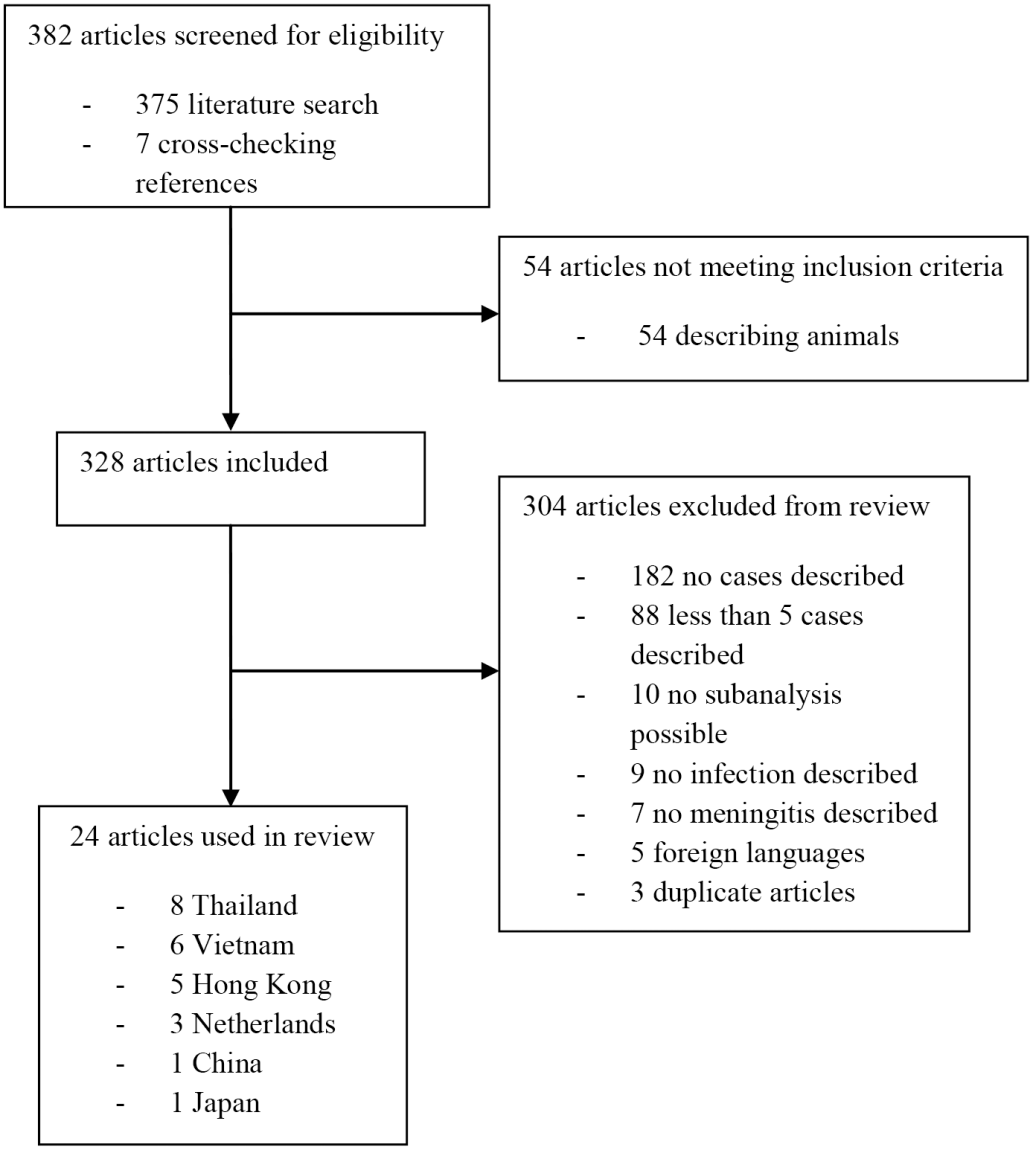
Flow-chart systematic review *Streptococcus suis* meningitis.

In total, 382 articles were screened for eligibility (375 by searching the databases and 7 by cross-checking references) (Fig 1). 54 articles did not meet the inclusion criteria as they described *S*. *suis* infection in animals. 304 articles were excluded from the review as no cases were described (183 articles), reporting less than 5 cases (88 articles), no sub-analysis possible for *S*. *suis* meningitis (10 articles), no *S*. *suis* infection described (9 articles), no meningitis described (7 articles), foreign language (5 articles) and duplicate articles (3 articles). The 24 articles included in the review described 913 patients [7, 8, 10, 12–32]. The number of included patients per study varied between 5 and 151 (median 21). The median described time-period of the studies was 6 years (ranging from 1 to 23 years). Studies were performed in Thailand (8 studies), Vietnam (6 studies), Hong Kong (5 studies), the Netherlands (3 studies), China (1 study) and Japan (1 study). Studies composed 10 single center studies, 4 multi-center studies and 10 nationwide studies. 11 studies included patients prospectively and 13 were retrospective studies.

### Clinical characteristics

The pooled mean age was 48.8 years (SD 3.9, reported in 715 cases) and 581 of 711 patients (82%, 95% CI 79–85%) were male (Table 1). Predisposing factors consisted of exposure to pig or pork in 395 of 648 patients (61%, 95% CI 57–65%), alcoholism in 60 of 322 patients (19%, 95% CI 15–23%), diabetes mellitus in 11 of 209 patients (5%, 95% CI 2–8%), cancer in 5 of 85 patients (6%, 95% CI 1–11%), splenectomy in 5 of 507 patients (1%, 95% CI 0–2%) and immunosuppressive medication in 2 of 593 patients (0.3%, 95% CI 0–0.8%).

**Table 1:** Clinical characteristics of patients with *S*. *suis* meningitis.

		**n/**N* **(%)**
**Age** ^a^ ^b^		48.8 (SD 3.9)
**Male**		581/711 (82%)
**Predisposing factors**		
Alcoholism		60/322 (19%)
Diabetes mellitus		11/209 (5%)
Splenectomy		5/507 (1%)
Immunosuppressive medication		2/601 (0.3%)
Cancer		5/85 (6%)
Exposure to pigs/pork		395/648 (61%)
**Clinical presentation**		
Skin injury in the presence of pig/pork contact		78/384 (20%)
Headache		429/451 (95%)
Fever		514/528 (97%)
Neck stiffness		462/496 (93%)
Altered consciousness		35/113 (31%)
Classic meningitis triad^1^		4/43 (9%)
Nausea/vomiting		210/321 (65%)
**Blood characteristics**		
Leukocytes^a^ ^c^		17.4 (SD 0.9)
Thrombocytes^a^ ^d^		166.3 (SD 19.1)
**Cerebrospinal fluid characteristics**		
Leukocytes/mm3 ^a^ ^e^		1920 (SD 757)
Protein (g/dL)^a^ ^f^		2.4 (SD 0.8)
Glucose (mmol/L)^a^ ^g^		1.09 (SD 0.60)
**Positive cultures**		
Cerebrospinal fluid		758/913 (83%)
Blood		288/435 (66%)
**Adjunctive dexamethasone**		157/300 (52%)
**Outcome**		
Death		17/581 (3%)
Hearing loss		259/489 (53%)
Other sequelae		35/286 (12%)
Full recovery		116/320 (36%)

N* number of patients in whom the symptom was reported

^1^Triad of fever, neck stiffness and altered consciousness

^a^Means are recalculated from means and medians

^b^Reported in 715 cases

^c^Reported in 322 cases

^d^Reported in 213 cases

^e^Reported in 395 cases

^f^Reported in 380 cases

^g^Reported in 177 cases

The clinical presentation of *S*. *suis* meningitis was characterized by fever in 514 of 528 patients (97%, 95% CI 96–98%), headache in 429 of 451 patients (95%, 95% CI 93–97%), neck stiffness in 462 of 496 patients (93%, 95% CI 91–95%), an altered consciousness in 35 of 113 patients (31%, 95% CI 23–39%) and nausea or vomiting in 210 of 321 patients (65%, 95% CI 60–70%). The classic meningitis triad of fever, neck stiffness and altered consciousness was present in 4 out of 43 patients (9%, 95% CI 0–18%) [2]. Skin injury in the presence of pig/pork contact was present in 78 of 384 patients (20%, 95% CI 16–24%).

### Ancillary investigations

The mean blood leukocyte count was 17.4 x 10^9^/L (SD 0.9, reported in 322 cases). The mean blood thrombocyte count was 166.3 x 10^9^/L (SD 19.1, reported in 213 cases). The mean cerebrospinal fluid (CSF) leukocyte count was 1920/mm^3^ (SD 757); it was reported in 395 patients and abnormal in all 913 patients. The mean CSF protein was 2.4 g/L (SD 0.8, reported in 380 patients) and the mean CSF glucose was 1.09 mmol/L (SD 0.60, reported in 177 patients).

Data on cerebrospinal fluid cultures were reported in all 913 patients, and were positive in 758 (83%, 95% CI 81–85%). Blood cultures were positive in 288 of 435 cases (66%, 95% CI 62–70%). Results of cranial CT were noted in 3 studies describing 27 patients [23, 28, 32] and consisted of cerebral edema in 8 of 27 patients (30%, 95% CI 10–50%).

### Treatment

The majority of patients was treated with ceftriaxone (250 patients) or penicillin (102 patients) monotherapy; no antibiotic resistance for these antibiotics was found in the 182 cases where the resistance pattern was determined. Antibiotic resistance for tetracycline was reported in 2 studies [7, 33]. In some studies, patients were treated with either penicillin or ceftriaxone (101 patients), but the exact number of patients receiving either treatment was not reported [23, 24, 29, 31]. In 454 patients, the type of antibiotic treatment was unknown. 157 of 300 patients (52%, 95% CI 44–60%) received adjunctive dexamethasone. The majority of these patients were included in a randomized controlled trial in which 71 patients received adjunctive dexamethasone and 69 patients received placebo [7]. In the other studies, dexamethasone was given at the discretion of the treating physician.

### Outcome

The case fatality rate was 2.9% (17 of 581 patients, 95% CI 1.9–3.9%) and 116 of 320 patients (36%, 95% CI 31–41%) recovered without sequelae. An association between dexamethasone and death could not be established because numbers of patients who died were small. Data from the RCT showed no patients died in dexamethasone group versus three in the placebo group [34]. Hearing loss was present in 259 of 489 patients (53%, 95% CI 49–57%). 68 of these patients were screened at admission for hearing loss and this was present in 60 patients (88%, 95% CI 80–96%). According to a study describing 41 patients with hearing loss in *S*. *suis* meningitis, 38 had hearing loss on admission and 3 developed hearing loss during admission [23]. Another study described 16 patients with *S*. *suis* meningitis and hearing loss, with hearing loss persisting in 7 patients (44%) [28]. Other neurological sequelae were present in 35 of 286 patients (12%, 95% CI 8–16%) and consisted of ataxia in 19 patients, cognitive impairment in 2, tinnitus in 2, and were not specified in 12.

A randomized controlled trial showed that dexamethasone was significantly associated with a reduction in hearing loss in at least one ear (38% to 12%, p = 0.003) and a reduction in severe (>80 dB) hearing loss (odds ratio 0.23 [95% CI, 0.06–0.78]), using a multivariate analysis including age >50 and CSF bacterial load [7]. A recent case series from the Netherlands showed that despite dexamethasone treatment 6 out of 7 patients with *S*. *suis* meningitis had hearing loss upon discharge [32].

## Discussion

Meningitis is the most frequently described presentation of *S*. *suis* infection, occurring in approximately 50–60% of reported *S*. *suis* infected patients [9]. Despite the geographical distribution, there were no significant differences for clinical presentation and outcome in *S*. *suis* meningitis between the different studies and low-/high-income countries. In our meta-analysis the main risk factor for *S*. *suis* meningitis was exposure to pigs or raw pork. This confirms the findings by a single center case-control study from Vietnam of 101 patients with *S*. *suis* infection which showed an odds ratio of 6.33 for occupations related to pigs [16]. Another previously reported potential risk factor was alcoholism, which we identified in 16% of patients. Alcoholism was not an independent risk factor for contracting *S*. *suis* meningitis when corrected for other predisposing factors in Vietnam [16]. However, alcoholism has been associated with an increased risk of infection in general and of an unfavorable outcome of bacterial meningitis [35].

Skin injury in the presence of pig/pork contact was described in 20% of the cases, which is similar to the previously observed 25% skin injuries in all types of *S*. *suis* infections [9]. *S*. *suis* may directly pass into the blood stream after exposure to pigs or pork in the presence of skin injuries, even without visible wound infection [10, 16, 36]. Patients with an increased risk of infection, e.g. because of splenectomy or use of immunosuppressive medication, should avoid direct pig or pork contact when skin lesions, particularly on the hands, are present. Skin protection has been suggested to reduce the incidence of *S*. *suis* infection [16].

Direct exposure to pigs or pork was described for 61% of meningitis cases. Direct pig exposure was documented in the majority of the European cases of *S*. *suis* infection, but was reported in less than half of the Asian cases, suggesting that other mechanisms may be involved in those patients [16]. A recent study showed that the gastro-intestinal tract is an entry site for *S*. *suis* [37], supporting the epidemiological evidence that ingestion of *S*. *suis* contaminated food is a risk factor for infection [9, 16, 38].

The sensitivity of the classic triad of bacterial meningitis consisting of fever, neck stiffness, and altered mental status was low (9%). This was mainly due to the low frequency of altered mental status, since other symptoms and signs of bacterial meningitis were present in a large proportion of patients. In patients with a history of regular pig exposure or pork consumption, hearing loss and these symptoms, meningitis due to *S*. *suis* should be suspected, and CSF examination should be performed to get diagnostic certainty [3].

We found that the mortality of *S*. *suis* meningitis was low (3%), especially when compared to pneumococcal meningitis (20%) and *Listeria monocytogenes* meningitis (36%) [39, 40]. The mortality rate was also lower than reported for general invasive infection caused by *S*. *suis* (13%) [12]. The difference between mortality in *S*. *suis* meningitis and other types of *S*. *suis* infection (such as sepsis) has been noted before [6, 8, 9, 19], but the mechanism causing this difference needs to be further elucidated [9]. Similar differences between meningitis and sepsis case fatality rates have been reported for invasive meningococcal disease [41].

The mortality rate was low but many surviving patients have sequelae. The most common sequel is hearing loss occurring in 53% of the patients; variable rates of hearing loss have been reported in other types of bacterial meningitis, with 8% in meningococcal meningitis and 22% in pneumococcal meningitis [2]. Hearing loss in *S*. *suis* meningitis may be a presenting symptom or develop during admission [23], and does not always persist [28]. Different hypotheses for hearing loss in *S*. *suis* meningitis are described in the literature such as direct infection of the auditory nerve and suppurative labyrinthitis [42]. For patients with meningitis in whom *S*. *suis* is identified, it is important to consult the otorhinolaryngologist early in the clinical course for audiometry and evaluate whether cochlear implantation is possible [43].

Dexamethasone has been shown to decrease mortality in pneumococcal meningitis and to decrease hearing loss and neurological sequelae in all bacterial meningitis cases [44, 45]. For *S*. *suis* meningitis, an effect on mortality has not been established [34]. One randomized controlled trial on dexamethasone in bacterial meningitis, performed in Vietnam, included a substantial number of *S*. *suis* meningitis [34]. A subsequent analysis of all *S*. *suis* patients showed dexamethasone reduced hearing loss in a multivariate analysis [7]. As a recent case-series showed, hearing loss is still observed in patients treated with dexamethasone [32], additional randomized clinical trials on the effect of dexamethasone in *S*. *suis* meningitis would be desirable to further evaluate whether there is a benefit. However, it is unlikely such a trial is going to be performed for practical and financial reasons. Based on the available evidence, dexamethasone treatment in regions with high rates of *S*. *suis* as cause of meningitis appears reasonable to potentially reduce the very high rate of post-meningitic hearing impairment.

This review has several limitations. First, most included studies show a selection bias due to a retrospective character. A recent study showed evidence of publication bias in *S*. *suis* meningitis [9]. *S*. *suis* meningitis is probably underreported, and often in numbers of less than 5 cases, which was an exclusion criterion for this study. Second, reporting of clinical characteristics, ancillary investigations and outcome was highly diverse between the included studies. We have presented the total number of patients in whom the specific characteristic was reported, but we could not perform a risk factor analysis due to heterogeneity in data. Third, cases of *S*. *suis* meningitis might have been missed due to a negative CSF culture caused by pre-treatment with antibiotics.

In conclusion, *S*. *suis* meningitis is predominantly seen in men after contact with pigs or pork and is endemic in pig rearing and pork consuming countries such as Vietnam, Thailand and China. The typical clinical presentation consists of hearing loss, fever, headache and neck stiffness, and skin injury in the presence of pig/pork contact is present in 20% of the cases. Although the mortality of *S*. *suis* meningitis is low compared to *S*. *suis* infection in general and other causes of bacterial meningitis, 53% of patients end up with hearing loss.

## Supporting Information

S1 Checklist PRISMA Checklist. (DOC)

S1 Flowchart PRISMA Flowchart. (DOC)

## References

[1] Brouwer MC, Tunkel AR, van de Beek D. Epidemiology, diagnosis, and antimicrobial treatment of acute bacterial meningitis. Clin Microbiol Rev. 2010;23: 467–492. 10.1128/CMR.00070-09 20610819 PMC2901656

[2] van de Beek D, de Gans J, Spanjaard L, Weisfelt M, Reitsma JB, Vermeulen M. Clinical features and prognostic factors in adults with bacterial meningitis. N Engl J Med. 2004;351: 1849–1859. 15509818 10.1056/NEJMoa040845

[3] Faucqueur B, Proust J. [Streptococcus suis meningitis. An occupational disease]. Presse Med. 1983;12: 1821. 6224200

[4] Lun ZR, Wang QP, Chen XG, Li AX, Zhu XQ. Streptococcus suis: an emerging zoonotic pathogen. Lancet Infect Dis. 2007;7: 201–209. 17317601 10.1016/S1473-3099(07)70001-4

[5] Wang G, Zeng YL, Liu HY, Xiong ZY. An outbreak of Streptococcus suis in Chengdu, China. Int J Clin Pract. 2007;61: 1056–1057. 17504369 10.1111/j.1742-1241.2007.01369.x

[6] Yang WZ, Yu HJ, Jing HQ, Xu JG, Chen ZH, Zhu XP, et al [An outbreak of human Streptococcus suis serotype 2 infections presenting with toxic shock syndrome in Sichuan, China]. Zhonghua Liu Xing Bing Xue Za Zhi. 2006;27: 185–191. 16792880

[7] Mai NT, Hoa NT, Nga TV, Linh le D, Chau TT, Sinh DX, et al Streptococcus suis meningitis in adults in Vietnam. Clin Infect Dis. 2008;46: 659–667. 10.1086/527385 19413493

[8] Yu H, Jing H, Chen Z, Zheng H, Zhu X, Wang H, et al Human Streptococcus suis outbreak, Sichuan, China. Emerg Infect Dis. 2006;12: 914–920. 16707046 10.3201/eid1206.051194 PMC3373052

[9] Huong VT, Ha N, Huy NT, Horby P, Nghia HD, Thiem VD, et al Epidemiology, clinical manifestations, and outcomes of Streptococcus suis infection in humans. Emerg Infect Dis. 2014;20: 1105–1114. 10.3201/eid2007.131594 24959701 PMC4073838

[10] Arends JP, Zanen HC. Meningitis caused by Streptococcus suis in humans. Rev Infect Dis. 1988;10: 131–137. 3353625 10.1093/clinids/10.1.131

[11] Hozo SP, Djulbegovic B, Hozo I. Estimating the mean and variance from the median, range, and the size of a sample. BMC Med Res Methodol. 2005;5: 13 15840177 10.1186/1471-2288-5-13 PMC1097734

[12] Fongcom A, Pruksakorn S, Netsirisawan P, Pongprasert R, Onsibud P. Streptococcus suis infection: a prospective study in northern Thailand. Southeast Asian J Trop Med Public Health. 2009;40: 511–517. 19842437

[13] Ho Dang Trung N, Le Thi Phuong T, Wolbers M, Nguyen Van Minh H, Nguyen Thanh V, Van MP, et al Aetiologies of central nervous system infection in Viet Nam: a prospective provincial hospital-based descriptive surveillance study. PLoS One. 2012;7: e37825 10.1371/journal.pone.0037825 22662232 PMC3360608

[14] Navacharoen N, Chantharochavong V, Hanprasertpong C, Kangsanarak J, Lekagul S. Hearing and vestibular loss in Streptococcus suis infection from swine and traditional raw pork exposure in northern Thailand. J Laryngol Otol. 2009;123: 857–862. 10.1017/S0022215109004939 19275779

[15] Nga TV, Nghia HD, Tu le TP, Diep TS, Mai NT, Chau TT, et al Real-time PCR for detection of Streptococcus suis serotype 2 in cerebrospinal fluid of human patients with meningitis. Diagn Microbiol Infect Dis. 2011;70: 461–467. 10.1016/j.diagmicrobio.2010.12.015 21767702 PMC3146703

[16] Nghia HD, Tu le TP, Wolbers M, Thai CQ, Hoang NV, Nga TV, et al Risk factors of Streptococcus suis infection in Vietnam. A case-control study. PLoS One. 2011;6: e17604 10.1371/journal.pone.0017604 21408132 PMC3050921

[17] Schultsz C, Jansen E, Keijzers W, Rothkamp A, Duim B, Wagenaar JA, et al Differences in the population structure of invasive Streptococcus suis strains isolated from pigs and from humans in The Netherlands. PLoS One. 2012;7: e33854 10.1371/journal.pone.0033854 22563452 PMC3341392

[18] Taylor LE, Foont JA, DeLong AK, Wurcel A, Linas BP, Chapman S, et al The spectrum of undiagnosed hepatitis C virus infection in a US HIV clinic. AIDS Patient Care STDS. 2014;28: 4–9. 10.1089/apc.2013.0130 24428794 PMC3894677

[19] Wertheim HF, Nghia HD, Taylor W, Schultsz C. Streptococcus suis: an emerging human pathogen. Clin Infect Dis. 2009;48: 617–625. 10.1086/596763 19191650

[20] Chang B, Wada A, Ikebe T, Ohnishi M, Mita K, Endo M, et al Characteristics of Streptococcus suis isolated from patients in Japan. Jpn J Infect Dis. 2006;59: 397–399. 17186962

[21] Ip M, Fung KS, Chi F, Cheuk ES, Chau SS, Wong BW, et al Streptococcus suis in Hong Kong. Diagn Microbiol Infect Dis. 2007;57: 15–20. 16860513 10.1016/j.diagmicrobio.2006.05.011

[22] Ma E, Chung PH, So T, Wong L, Choi KM, Cheung DT, et al Streptococcus suis infection in Hong Kong: an emerging infectious disease? Epidemiol Infect. 2008;136: 1691–1697. 10.1017/S0950268808000332 18252026 PMC2870785

[23] Rusmeechan S, Sribusara P. Streptococcus suis meningitis: the newest serious infectious disease. J Med Assoc Thai. 2008;91: 654–658. 18672627

[24] Wangsomboonsiri W, Luksananun T, Saksornchai S, Ketwong K, Sungkanuparph S. Streptococcus suis infection and risk factors for mortality. J Infect. 2008;57: 392–396. 10.1016/j.jinf.2008.08.006 18835496

[25] Chau PY, Huang CY, Kay R. Streptococcus suis meningitis. An important underdiagnosed disease in Hong Kong. Med J Aust. 1983;1: 414–416, 417 6835158

[26] Donsakul K, Dejthevaporn C, Witoonpanich R. Streptococcus suis infection: clinical features and diagnostic pitfalls. Southeast Asian J Trop Med Public Health. 2003;34: 154–158. 12971528

[27] Hui AC, Ng KC, Tong PY, Mok V, Chow KM, Wu A, et al Bacterial meningitis in Hong Kong: 10-years' experience. Clin Neurol Neurosurg. 2005;107: 366–370. 16023529 10.1016/j.clineuro.2004.10.006

[28] Kay R, Cheng AF, Tse CY. Streptococcus suis infection in Hong Kong. QJM. 1995;88: 39–47. 7894987

[29] Suankratay C, Intalapaporn P, Nunthapisud P, Arunyingmongkol K, Wilde H. Streptococcus suis meningitis in Thailand. Southeast Asian J Trop Med Public Health. 2004;35: 868–876. 15916083

[30] Vilaichone RK, Vilaichone W, Nunthapisud P, Wilde H. Streptococcus suis infection in Thailand. J Med Assoc Thai. 2002;85 Suppl 1: S109–117. 12188400

[31] Wangkaew S, Chaiwarith R, Tharavichitkul P, Supparatpinyo K. Streptococcus suis infection: a series of 41 cases from Chiang Mai University Hospital. J Infect. 2006;52: 455–460. 16690131 10.1016/j.jinf.2005.02.012

[32] van Samkar A, Brouwer MC, Schultsz C, van der Ende A, van de Beek D. Streptococcus suis meningitis in the Netherlands. J Infect. 2015 10.1016/j.jinf.2015.07.001 26165610

[33] Ma Z, Yu L, Zhou H, Liu T, Xu B, Ma F, et al Identification of novel genes expressed during host infection in Streptococcus equi ssp. zooepidemicus ATCC35246. Microb Pathog. 2015;79: 31–40. 10.1016/j.micpath.2015.01.004 25595678

[34] Nguyen TH, Tran TH, Thwaites G, Ly VC, Dinh XS, Ho Dang TN, et al Dexamethasone in Vietnamese adolescents and adults with bacterial meningitis. N Engl J Med. 2007;357: 2431–2440. 18077808 10.1056/NEJMoa070852

[35] Weisfelt M, de Gans J, van der Ende A, van de Beek D. Community-acquired bacterial meningitis in alcoholic patients. PLoS One. 2010;5: e9102 10.1371/journal.pone.0009102 20161709 PMC2817003

[36] Ferrando ML, de Greeff A, van Rooijen WJ, Stockhofe-Zurwieden N, Nielsen J, Wichgers Schreur PJ, et al Host-pathogen Interaction at the Intestinal Mucosa Correlates With Zoonotic Potential of Streptococcus suis. J Infect Dis. 2015;212: 95–105. 10.1093/infdis/jiu813 25525050 PMC4462715

[37] Ferrando ML, de Greeff A, van Rooijen WJ, Stockhofe-Zurwieden N, Nielsen J, Wichgers Schreur PJ, et al Host-pathogen Interaction at the Intestinal Mucosa Correlates With Zoonotic Potential of Streptococcus suis. J Infect Dis. 2014. 10.1093/infdis/jiu813 PMC4462715 25525050

[38] Takeuchi D, Kerdsin A, Pienpringam A, Loetthong P, Samerchea S, Luangsuk P, et al Population-based study of Streptococcus suis infection in humans in Phayao Province in northern Thailand. PLoS One. 2012;7: e31265 10.1371/journal.pone.0031265 22363601 PMC3283636

[39] Brouwer MC, Heckenberg SG, de Gans J, Spanjaard L, Reitsma JB, van de Beek D. Nationwide implementation of adjunctive dexamethasone therapy for pneumococcal meningitis. Neurology. 2010;75: 1533–1539. 10.1212/WNL.0b013e3181f96297 20881273

[40] Koopmans MM, Brouwer MC, Bijlsma MW, Bovenkerk S, Keijzers W, van der Ende A, et al Listeria monocytogenes sequence type 6 and increased rate of unfavorable outcome in meningitis: epidemiologic cohort study. Clin Infect Dis. 2013;57: 247–253. 10.1093/cid/cit250 23592828

[41] Heckenberg SG, de Gans J, Brouwer MC, Weisfelt M, Piet JR, Spanjaard L, et al Clinical features, outcome, and meningococcal genotype in 258 adults with meningococcal meningitis: a prospective cohort study. Medicine (Baltimore). 2008;87: 185–192. 18626301 10.1097/MD.0b013e318180a6b4

[42] Tan JH, Yeh BI, Seet CS. Deafness due to haemorrhagic labyrinthitis and a review of relapses in Streptococcus suis meningitis. Singapore Med J. 2010;51: e30–33. 20358139

[43] Barbosa MH, Felix F, Ribeiro MG, Tomita S, Pinheiro C, Baptista MM. Profile of patients assessed for cochlear implants. Braz J Otorhinolaryngol. 2014;80: 305–310. 10.1016/j.bjorl.2014.05.011 25183180 PMC9444620

[44] van de Beek D, Farrar JJ, de Gans J, Mai NT, Molyneux EM, Peltola H, et al Adjunctive dexamethasone in bacterial meningitis: a meta-analysis of individual patient data. Lancet Neurol. 2010;9: 254–263. 10.1016/S1474-4422(10)70023-5 20138011 PMC2835871

[45] Brouwer MC, McIntyre P, Prasad K, van de Beek D. Corticosteroids for acute bacterial meningitis. Cochrane Database Syst Rev. 2013;6: CD004405 10.1002/14651858.CD004405.pub4 23733364

