# Supplementary material for: Streptococcus suis Meningitis: A Systematic Review and Meta-analysis
Source: PLoS Negl Trop Dis. 2015 Oct 27;9(10):e0004191. doi: 10.1371/journal.pntd.0004191 (PMC4624688; doi:10.1371/journal.pntd.0004191)
Supplement: S1 Flowchart — (DOC) [file pntd.0004191.s002.doc]

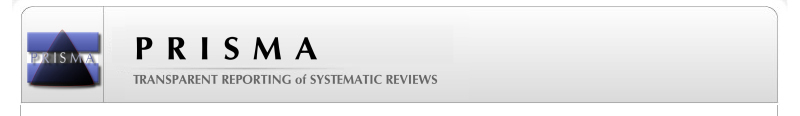
**PRISMA 2009 Flow Diagram**

**Screening**

**Included**

**Eligibility**

**Identification**

Records identified through database searching
(n = 375 )

Additional records identified through other sources
(n = 7 )

Records after duplicates removed
(n = 3)

Records screened
(n = 379 )

Records excluded
(n = 54)

Full-text articles assessed for eligibility
(n = 325 )

Full-text articles excluded, with reasons
(n = 301 )

- 182 no cases described

- 88 less than 5 patients

- 10 no subanalysis possible

- 9 no infection described

- 7 no meningitis described

- 5 foreign languages

Studies included in qualitative synthesis
(n = 24 )

Studies included in quantitative synthesis (meta-analysis)
(n = 24 )
